# Exploring the predictive value of structural covariance networks for the diagnosis of schizophrenia

**DOI:** 10.3389/fpsyt.2025.1570797

**Published:** 2025-06-09

**Authors:** Clara S. Vetter, Annika Bender, Dominic B. Dwyer, Max Montembeault, Anne Ruef, Katharine Chisholm, Lana Kambeitz-Ilankovic, Linda A. Antonucci, Stephan Ruhrmann, Joseph Kambeitz, Marlene Rosen, Theresa Lichtenstein, Anita Riecher-Rössler, Rachel Upthegrove, Raimo K. R. Salokangas, Jarmo Hietala, Christos Pantelis, Rebekka Lencer, Eva Meisenzahl, Stephen J. Wood, Paolo Brambilla, Stefan Borgwardt, Peter Falkai, Alessandro Bertolino, Nikolaos Koutsouleris, PRONIA Consortium

**Affiliations:** ^1^ Department of Psychiatry and Psychotherapy, Ludwig-Maximilian-University, Munich, Germany; ^2^ Munich Center of Machine Learning (MCML), Munich, Germany; ^3^ Helmholtz Association - Munich School for Data Science (MUDS), Munich, Germany; ^4^ Centre for Youth Mental Health, University of Melbourne, Melbourne, VIC, Australia; ^5^ The National Centre of Excellence for Youth Mental HealthOrygen, Melbourne, VIC, Australia; ^6^ Douglas Research Centre and Department of Psychiatry, McGill University, Montréal, QC, Canada; ^7^ School of Psychology, University of Sussex, Brighton, United Kingdom; ^8^ Department of Psychiatry and Psychotherapy, Faculty of Medicine and University Hospital of Cologne, University of Cologne, Germany, Cologne, Germany; ^9^ Department of Translational Biomedicine and Neuroscience (DiBraiN), University of Bari “Aldo Moro”, Bari, Italy; ^10^ Faculty of Medicine, University of Basel, Basel, Switzerland; ^11^ Department of Psychiatry, University of Oxford, Oxford, United Kingdom; ^12^ Department of Psychiatry, University of Turku, Turku, Finland; ^13^ Melbourne Neuropsychiatry Centre, Department of Psychiatry, University of Melbourne, Melbourne, VIC, Australia; ^14^ NorthWestern Mental Health, Royal Melbourne Hospital, Melbourne, VIC, Australia; ^15^ Institute for Translational Psychiatry, University of Muenster, Muenster, Germany; ^16^ Department of Psychiatry and Psychotherapy, University of Lübeck, Lübeck, Germany; ^17^ Department of Psychiatry and Psychotherapy, Medical Faculty, Heinrich-Heine University, Düsseldorf, Germany; ^18^ School of Psychology, University of Birmingham, Birmingham, United Kingdom; ^19^ Department of Neurosciences and Mental Health, Fondazione IRCCS Ca’ Granda Ospedale Maggiore Policlinico, University of Milan, Milan, Italy; ^20^ Department of Pathophysiology and Transplantation, University of Milan, Milan, Italy; ^21^ Department of Pathophysiology and Transplantation, Max Planck Institute for Psychiatry, Munich, Germany; ^22^ Department of Psychosis Studies, Institute of Psychiatry, Psychology Neuroscience, King’s College London, London, United Kingdom

**Keywords:** precision psychiatry, schizophrenia, structural covariance, machine learning, neuroimaging, brain connectivity

## Abstract

**Introduction:**

Schizophrenia is a psychiatric disorder hypothesized to result from disturbed brain connectivity. Structural covariance networks (SCN) describe the shared variation in morphological properties emerging from coordinated neurodevelopmental processes, This study evaluates the potential of SCNs as diagnostic biomarker for schizophrenia.

**Methods:**

We compared the diagnostic value of two SCN computation methods derived from regional gray matter volume (GMV) in 154 patients with a diagnosis of first episode psychosis or recurrent schizophrenia (PAT) and 366 healthy control individuals (HC). The first method (REF-SCN) quantifies the contribution of an individual to a normative reference group’s SCN, and the second approach (KLS-SCN) uses a symmetric version of Kulback-Leibler divergence. Their diagnostic value compared to regional GMV was assessed in a stepwise analysis using a series of linear support vector machines within a nested cross-validation framework and stacked generalization, all models were externally validated in an independent sample (N_PAT_=71, N_HC_=74), SCN feature importance was assessed, and the derived risk scores were analyzed for differential relationships with clinical variables.

**Results:**

We found that models trained on SCNs were able to classify patients with schizophrenia and combining SCNs and regional GMV in a stacked model improved training (balanced accuracy (BAC)=69.96%) and external validation performance (BAC=67.10%). Among all unimodal models, the highest discovery sample performance was achieved by a model trained on REF-SCN (balanced accuracy (BAC=67.03%). All model decisions were driven by widespread structural covariance alterations involving the somato-motor, default mode, control, visual, and the ventral attention networks. Risk estimates derived from KLS-SCNs and regional GMV, but not REF-SCNs, could be predicted from clinical variables, especially driven by body mass index (BMI) and affect-related negative symptoms.

**Discussion:**

These patterns of results show that different SCN computation approaches capture different aspects of the disease. While REF-SCNs contain valuable information for discriminating schizophrenia from healthy control individuals, KLS-SCNs may capture more nuanced symptom-level characteristics similar to those captured by PCA of regional GMV.

## 1 Introduction

Schizophrenia has been conceptualized as a neurodevelopmental disorder that features structural deficits across numerous brain regions (1–5), such as widespread gray matter loss and cortical thinning (6–8), which are thought to reflect synaptic density alterations (reviewed in e.g., Howes et al. (2023) (9)). Together with evidence for disrupted neuronal communication underlying the diverse phenotypes of the disorder, these findings have given rise to the “dysconnectivity hypothesis” of schizophrenia, according to which symptoms stem from impaired anatomical and functional connectivity between brain regions rather than only region-specific changes (10–13). Structural changes and altered structural and functional connectivity may be interrelated and associated with similar neurodevelopmental genetic, prenatal, and environmental factors (6–8, 14, 15).

Systems-level alterations in brain organization can also be observed in networks based on brain structure covariance, which describe the shared variation in morphological properties, e.g., gray matter volume (GMV), cortical thickness, surface area, and gyrification of brain regions across a population (16–20). Structural covariance networks (SCNs) emerge from coordinated neurodevelopmental processes (20–23), which reflect anatomical connectivity (24), mutually trophic influences (25), and common experience-driven plasticity (26). SCNs demonstrate small-world organization (27), a well-studied, graph-theoretical property of brain networks, and are organized in modules overlapping with functional domains (28). Structural covariance has been shown to be highly heritable, to change significantly across the lifespan (21–23, 29), and to be linked to IQ (23) and brain disorders, including schizophrenia (30–35), autism (36), attention deficit hyperactivity disorder (ADHD) (37), and Alzheimer’s disease (38).

Studies investigating the topology of structural covariance networks (SCN) in schizophrenia with metrics from graph theory (for an overview, see (39)) suggest qualitative and quantitative differences between SCNs of schizophrenia patients and controls (33). These differences may emerge from alterations of maturational trajectories (40, 41) before the onset of the disorder during (pre-)adolescence and aggravate with disease progression (42, 43). While definitive results from graph theoretical analyses of SCN in schizophrenia regarding implicated regions are still lacking, patterns of structural covariance between fronto-temporal, fronto-parietal and fronto-thalamic covariation seem to be altered (for a systematic review, see Prasad et al. (2022a) (32) and Prasad et al. (2022b) (33)).

The heterogeneity of clinical phenotypes of schizophrenia, may be reflected by variations in structural covariance, in line with the dysconnectivity hypothesis of schizophrenia. While Spreng et al. (34) found no relationship between positive and negative symptoms of psychosis, others report supporting findings (44–46). For example, structural covariance of the salience network has been negatively correlated with symptom severity in first episode psychosis patients (44). Further, dysconnectivity in the thalamus (45) and dorsolateral prefrontal cortex has been linked to various symptoms in schizophrenia patients (46). Additionally, treatment response to antipsychotic medication is related to the morphological reconfiguration of brain networks, i.e., structural covariance (47, 48). Jiang et al. (2022) (47) provided evidence of increased interregional covariance in antipsychotic medication responders compared to non-responders among first-episode schizophrenia patients suggesting that the reconfiguration of morphological architecture, i.e., structural covariance, induced by antipsychotic medication acts as a compensatory mechanism for cortical abnormalities. The heterogeneity in brain structural changes in schizophrenia may be explained by comorbidities and cooccurring medical conditions, such as obesity (49, 50) which is disproportionally frequent among schizophrenia patients (51). Obesity, with body mass index (BMI) being an often employed proxy measure, has been linked to changes in brain structure and connectivity (52–54) and structural covariance alterations (55). Further, both brain structure and structural covariance of the perigenual anterior cingulate cortex has been found to be a predictor of future weight gain (56).

SCNs are typically computed at the group-level by means of correlations (not covariance) across participants (27), and can, thus, only be used to identify group-level differences, making individual diagnosis and prognosis and the discovery of connectivity-based biomarkers impossible. Mapping individual SCN differences can facilitate finding clinical and genetic correlates (35, 43, 57) as well as neuroanatomical patient subtypes. In addition, relationships with clinical and environmental factors may exhibit clearer associations.

To date, few methods have been proposed to estimate individual-level SCNs, which differ with regard to the type and number of morphological properties considered, and whether a normative sample is used for computation (19, 57–65). Here, we compare a normative and a single-image-based approaches that both have frequently been reported. The first approach proposed by Saggar et al. (2015) (62) uses a large reference group of healthy control (HC) individuals to derive individual SCNs. This method defines an individual’s SCN as the difference between a group SCN computed across the reference group and the group SCN computed across the reference group plus the respective individual. This approach was also adopted by Drenthen et al. (2018, 2022) (66, 67). Similarly, Das et al. (2018) (57) proposed to compute an individual’s SCN as the difference between the group SCN of the diagnostic group of the participant and the SCN of the diagnostic group excluding the given individual. The second approach was introduced by Kong et al. (2014) (59) and defines an individual’s SCN computing the pairwise symmetric Kulback-Leibler divergence (KLS) between the probability density functions (PDF) of regional voxel-wise GMV. This method has since been adopted by several studies (58, 64, 68, 69), and most recently extended to the multimodal setting (63).

In this study, our primary objective was to assess the diagnostic validity of two alternative individual SCN estimation methods based on GMV in discriminating patients with schizophrenia from HC individuals. We opted for a simplified binary classification task and linear machine learning algorithms to enable a direct comparison of different feature computation techniques and direct model explainability, leveraging well-established structural brain changes in schizophrenia. To the best of our knowledge, no previous research has investigated the differential and complementary diagnostic value of SCNs derived using these two distinct approaches in comparison to region-of-interest (ROI)-GMV.

We hypothesized that machine learning models trained on individual SCNs would be superior in distinguishing patients with schizophrenia from HC individuals compared to models trained on ROI-GMV. Furthermore, we anticipated that integrating regional GMV and SCN models through a stacking-based approach would lead to enhanced performance, given that these modalities may capture distinct aspects of the disorder. Additionally, we conducted an analysis to identify the most influential regions and structural covariance features driving the classification decisions in the top-performing models.

To gain further insights into the clinical relevance of the feature modalities, we investigated whether the decision scores generated by the regional GMV and SCN models are sensitive to disease phenotypes and thus could explain different aspects of the well-known heterogeneity of schizophrenia. To achieve this, we evaluated the extent to which machine learning could predict decision/risk scores generated from our different SCN or GMV-based models using clinical and demographic variables.

## 2 Methods

### 2.1 Datasets for model training and validation

Model training and validation were performed using two independent datasets. The training dataset comprised 366 HC individuals and 154 patients with DSM-IV diagnosis of schizophrenia (see (70) for details). All participants were recruited at the Department of Psychiatry and Psychotherapy, Ludwig-Maximilian-University, Munich, Germany. Henceforth, the sample will be referred to as the MUC sample. The validation dataset was provided by the Mind Research Networks Center for Biomedical Research Excellence (COBRE; https://coins.trendscenter.org) and consisted of 71 patients with chronic schizophrenia (SCZ) and 74 HC individuals. Patients were diagnosed using the Structured Clinical Interview for DSM-IV (SCID (71)). For more information on study procedures, see http://fcon_1000.projects.nitrc.org/indi/retro/cobre.html). The COBRE dataset is distributed under the Creative Commons License, and all participants provided written informed consent according to the ethics review board protocols of the University of New Mexico (UNM) (72). We evaluated demographic differences between diagnostic groups within the two samples using Fisher’s exact test or the Wilcoxon rank sum test with continuity correction. These tests were chosen since not all variables were normally distributed. Additionally, differences between discovery and validation samples were analyzed using the same methods. See **Table 1** for detailed sociodemographic and clinical characteristics of both samples.

**Table 1:** Sociodemographic and clinical characteristics of the Munich (MUC) sample (N=520) and COBRE sample (N=145).

Variable	MUC sample			COBRE sample			
	HC indiv. N=366	Patients N=154	P (t/ W, df)	HC indiv. N=74	Patients N=71	P (t/ W, df)	P (t/ W, df)
Mean age in years (SD)	33.6 (11.2)	30.8 (10.0)	.006^a^(2.77, 518)	35.8 (11.6)	38.1 (14.0)	.278^a^ (-1.09, 143)	<.001^a^ (-3.57, 206)
N sex female (%)	184 (50.3)	41 (26.6)	<.001^b^	23 (31.1)	14 (19.7)	.131^b^	<.001^b^
N left handedness (%)	47 (12.8)	N/A		3 (4.1)	12 (16.9)	.014^b^	N/A
Mean years in education (SD)	12.1(1.3)	N/A		14.4 (3.3)	13.0 (1.9)	.003^c^(3.08, 103.36)	N/A
Mean IQ (WASI) (SD)		N/A		108.4 (22.2)	100.4 (16.9)	.020^a^(2.35, 123.31)	N/A
N first-episode SCZ (%)		66 (42.9)			N/A		N/A
N recurrent SCZ (%)		88 (57.1)			71 (100)		
Mean N Hospitalizations (SD)		2.0 (2.1)			5.3 (5.5)		<.001^g^
Mean age at illness onset (SD)		25.4 (8.1)			21.1 (7.5)		<.001^h^
Mean illness duration in years(SD)		4.5 (7.1)			16.8 (13.0)		<.001^i^
Mean PANSS total score (SD)		51.6 (28.2)			58.7 (13.8)		.014 ^j^
Mean PANSS positive score (SD)		11.6 (7.7)			14.8 (4.8)		<.001^k^
Mean PANSS negative score (SD)		15.0 (9.7)			14.6 (4.8)		.692^l^
Mean PANSS general score (SD)		25.0 (15.5)			29.2 (8.5)		.010^a^ (-2.59, 213)
Mean dose CPZ equiv. (SD)		341.8 (373.8)			369.2 (306.0)		.575^a^ (-.56, 163)
Mean dose OLZ equiv. (SD)		N/A			15.2 (11.1)		N/A

a Student’s t-test.

b Fisher’s exact test.

c Welch’s test; HC, healthy control; df, degrees of freedom; SD, standard deviation; IQ, intelligence quotient, WASI, Wechsler Abbreviated Scale Intelligence; PANSS, Positive And Negative Syndrome Scale; SANS, Scale for the Assessment of Negative Symptoms; CPZ, clozapine, OLZ, olanzapine.

### 2.2 Reference sample characteristics for individual reference group-based SCNs (REF-SCN) construction

For the construction of the SCNs, 489 HC individuals drawn from the PRONIA study (Personalized Prognostic Tools for Early Psychosis Management; http://proniapredictors.eu/pronia/index.html) and 138 HC individuals from the OASIS-3 release (Online Access Series of Imaging Studies; https://www.oasis-brains.org) served as the reference sample. For the demographic information of the reference sample, see **Supplementary Table S2**.

### 2.3 Processing of structural MRI data

High-resolution three-dimensional T1-weighted images were acquired for all participants. MRI data acquisition parameters are detailed in S1.1. All T1-weighted images were processed using the open-source CAT12 toolbox (version r1207 (73), an extension of SPM12 (74). For more information on the preprocessing pipeline see S1.2. From the processed T1-weighted images, regional GMV was computed by summing the voxel-wise GMV values within each region-of-interest (ROI) of two cortical parcellation schemes based on the Schaefer atlas, which divided the cortex in 100 and 200 parcels, respectively (75). The derived regional GMV were used as input features (d=100 and d=200) to the ROI GMV-based ML models.

### 2.4 Structural covariance networks

We derived two types of SCNs following (1), reference group-based SCNs (REF-SCN) and (2) symmetric KL-divergence based SCNs (KLS-SCN) for the two brain parcellations with 100 and 200 parcels (75). A REF-SCN represented the contribution of an individual to the group SCN of the reference group (62), for details on the network computation see S1.3. Before deriving the REF-SCNs, we accounted for systematic differences between the images across the different sites of the PRONIA sample and the OASIS-3 cohort (see **Supplementary Methods**). In the KLS-SCNs, an edge is quantified as the symmetric KL-divergence between the probability density functions of voxel-wise GMV. The workflow is described in S1.4. From each SCN modality, we derived two different feature sets which served as input to the ML models, (a) the vectorized upper triangle of the individual connectivity matrices (i.e., edges, E), and (b) network metrics (M), specifically global efficiency, transitivity, eigenvector centrality, strength, and local efficiency (see S1.5).

Taken together, our multi-atlas approach resulted in two ROI-based feature sets and eight SCN-based feature sets for the subsequent multimodal machine learning (ML) classification models: ROI-GMV 100, ROI-GMV 200, REF-SCN-E 100, REF-SCN-E 200, REF-SCN-M 100, REF-SCN-M 200, KLS-SCN-E 100, KLS-SCN-E 200, KLS-SCN-M 100, KLS-SCN-M 200.

### 2.5 Machine learning classification pipelines: schizophrenia patients vs. healthy control individuals

To assess the diagnostic value of ROI GMV and SCNs for individual classification of schizophrenia patients, we trained a total of 20 unimodal models (for an overview, see **Supplementary Figure S3**). The models differed with respect to the type of input features (ROI GMV vs. REF-SCN-E vs. KLS-SCN-E vs. REF-SCN-M vs. KLS-SCN-M), the cortical parcellation used (100 vs. 200 parcels), and the type of dimensionality reduction employed in the ML pipeline and algorithm employed (feature selection using LASSO regularization vs. principal component analysis, PCA). While LASSO (L1) regularization and PCA both reduce the dimensionality of the feature set, the resulting low-dimensional features differ significantly in their interpretation. While LASSO regularization acts as a feature selection technique by removing redundant and irrelevant features, PCA extracts information across all features into new orthogonal variance components.

Additional preprocessing steps included in the ML model pipelines differed depending on the dimensionality reduction method used. In the LASSO condition, minimal preprocessing was used for all modalities including adjusting for age and sex effects by partial correlation analysis, followed by feature-wise standardization. The classification algorithm used was a L1-regularized hinge-loss support vector classifier with a dual solver (L1-SVC) implemented in LIBLINEAR (76). In the PCA condition, processing involved adjusting for age and sex effects, PCA with the number of principal components being optimized in the range of n_PC_ ∈ [5, 10, 15, 20, 25], followed by standardization. The classification algorithm used in this condition was a L2-regularized hinge-loss support vector classifier with a dual solver (L2-SVC) implemented in LIBLINEAR. SVC was chosen based on recent findings related to the superior scalability of linear vs. non-linear models in neuroimaging datasets considering the limited available sample sizes (77). To mitigate the effects of class imbalance during training, we adjusted the hyperplane coefficients of our model by giving higher importance to the minority class by multiplying class weights calculated as the inverse of class frequencies.

For each separate dimensionality reduction condition, the trained unimodal models were then combined by using their standardized prediction scores as input to L2-SVCs to create ROI-based, REF-SCN-based, KLS-SCN-based ‘meta-classifiers’ through stacked generalization. Stacked generalization is an ensemble learning approach, where the multi-source, second-level model uses the prediction outputs of the single-source, first-level models to predict the output labels (78). In the SCN-stacked models, edge and metric-based model scores were combined to create unifying SCN models for each of the SCN computation approaches. Finally, the standardized prediction scores of the four resulting meta-learners (again, for each dimensionality reduction condition separately) were used as input for a 3^rd^-level ROI&SCN-based L2-SVCs multimodal stacked classifier. This resulted in in total six 2^nd^ and two 3^rd^-level stacked generalization models (for an overview, see **Supplementary Figure S3**).

The ML pipelines, including preprocessing, were implemented in NeuroMiner (version 1.2, https://github.com/neurominer-git/NeuroMiner_1.2). A repeated nested pooled cross-validation (CV) design was used with 10 outer (CV_2_) folds, 10 CV_2_ permutations and 10 inner (CV_1_) folds without permutations. Nesting was done to completely isolate model performance evaluation which was performed at the CV_2_ level from hyperparameter optimization conducted at CV_1_ cycle. All preprocessing steps and the stacked generalization analyses without exception were also conducted within the identical cross-validation structure to avoid overfitting through double-dipping (79).

Balanced accuracy (BAC) was used as the criterion for hyperparameter optimization. To produce a final prediction for the CV_2_ test data, all models in each CV_2_ training partition were retrained at the optimal hyperparameter combination using the entire CV_2_ training data, where optimal performance was defined by the maximum average BAC across the CV_1_ test data. The retrained models were applied to the CV_2_ test data of the given CV_2_ partition, and their decision scores were averaged into an ensemble prediction. Finally, the ensembles were identified across the CV_2_ permutations in which the given test data was not in the CV_2_ training fold and their predictions were integrated into a grand mean average decision score and a final class membership prediction. For all models, the slack parameter *C* of the L1-SVC and L2-SVC was optimized across the values C={2^x^ ∈ ℤ:-6<=x<=4}.

The statistical significance of the models was tested using 1000 random label permutations (80). The training performance in terms of BAC, sensitivity, specificity and AUC of the 28 models was compared using the Quade test followed by *post hoc* pairwise two-sample t-tests, which were corrected for multiple comparisons using the false-discovery rate (81). Generalization was assessed in terms of BAC, sensitivity, specificity, and AUC when the model was applied to the COBRE dataset for external validation. Prior to external validation, the COBRE data was adjusted to the training dataset by subtracting the difference in regional mean GMV values between the samples.

Global explainability analyses were conducted for the best model in each modality for both feature extraction conditions. The contribution of features to a models’ decisions were measured by means of the cross-validation ratio, a measure of pattern element stability which computes the mean and standard error of all weight vectors across the entire nested cross-validation structure and is inspired by bootstrap ratio (82), and signed-based consistency, a measure of pattern element relevance and significance (83). For the SCN-E models, estimates of regional involvement scores were defined by how often a region was involved in a significantly contributing edge (equal to degree centrality (84) of regions in a feature importance network).

The degree of shared or complementary neuroanatomical information captured by the different modalities was quantified using pairwise Pearson correlations among the decision scores of the models (significance was determined at an FDR-corrected threshold of *P*=0.05). Additionally, we evaluated the similarity of relevant information within in the feature sets prior to model training (i.e., after the preprocessing steps of the model pipelines were performed outside of the CV framework), specifically focusing on the relationship of ROI-GMV-PCA and the SCN-E models. Mean Pearson correlations of the processed SCN-E features with the first ROI-GMV principal component (PC) were chosen as a measure for this analysis.

Finally, cohort effects on decision scores were tested using t-tests and simple linear regression models including age and sex as additional effects.

### 2.6 Clinical prediction models

To explore the unique clinical and demographic associations of SCN vs. GMV-based model’s decision scores, we employed linear support vector regressors (SVR) within a repeated nested CV framework, as described above. Models were built with NeuroMiner (version 1.2, https://github.com/neurominer-git/NeuroMiner_1.2). The full model pipelines and training setup are detailed in S1.7. The SVRs were trained to predict the imaging-based decision scores using the patients’ clinical and demographic features such as age, sex, handedness, body mass index (BMI), years of education, information about the illness course (duration, onset, hospitalizations, first-generation antipsychotic medication, duration of untreated psychosis), symptoms assessed using the Positive And Negative Syndrome Scale (PANSS (85) and the Scale for the Assessment of Negative Symptoms (SANS (86), drug usage (for a full list, see S1.7)) within the patient sample of the MUC sample. Similarity of feature importance between the models was assessed using Pearson correlations.

## 3 Results

### 3.1 Sociodemographic, clinical, and global anatomical results

In the MUC sample (used for training), patients and HC individuals differed significantly regarding age (*N_HC_=*366*, N_PAT_=*154*, t_Student’_*_s_ *(518*)=2.771, *P*=.006) and sex (Fisher’s exact test, *P*<.001; see **Table 2** for an overview of all sociodemographic data and significance tests). In the COBRE sample (used for external validation), patients exhibited decreased years of education (*t_Welch’s_* (103.36)=3.08, *P*=.003) and IQ (*t_Student’s_* (123.31)=2.35, *P*=.020) and increased left-handedness (Fisher’s exact test, *P*=.014; see **Table 1**) compared to HC individuals. Participants of the COBRE sample were significantly older than those of the MUC sample, and the participants of the samples additionally exhibited a significant difference in sex (%-females_MUC_ *=*43.27, %-females_COBRE_=25.52, Fisher’s exact test, *P*<.001). When comparing only the patients of the respective samples, significant differences were found in disease status since the MUC sample, in contrast to the COBRE sample, includes not only patients with a recurrent SCZ but also patients with a diagnosis of first-episode SCZ (42.90%), their number of hospitalizations (*W* (75)=-4794, P*<.001*), age at illness onset (*W* (145)=3859, *P*<.001), illness duration (*W* (89)=-7416, *P*<.001), total (*W* (216)=-2485, *P=*.014), positive symptom (*W* (203)=-3836, *P*<.001), and general PANSS score (*W* (213)=-2585, *P*=.01; see **Table 1**). The sociodemographic characteristics of the reference sample are depicted in **Supplementary Table S1**.

**Table 2:** Classification model performances.

	Training on MUC sample				Validation on COBRE sample			
Model	BAC (%)	Sens. (%)	Spec. (%)	AUC	BAC (%)	Sens. (%)	Spec. (%)	AUC
ROI-GMV 100 LASSO	64.50	62.34	66.67	.68	65.92	52.11	79.73	.69
ROI-GMV 200 LASSO	65.54	61.69	69.40	.69	61.61	39.44	83.78	.65
ROI-GMV stacker LASSO	63.82	62.34	65.30	.69	64.54	50.70	78.38	.68
ROI-GMV 100 PCA	62.33	59.09	65.57	.68	65.89	50.70	81.08	.71
ROI-GMV 200 PCA	64.67	61.04	68.31	.70	65.19	49.30	81.08	.69
ROI-GMV stacker PCA	64.04	61.70	66.39	.70	66.60	52.11	81.08	.71
REF-SCN-E 100 LASSO	61.51	59.09	63.93	.65	53.72	16.90	90.54	.48
REF-SCN-E 200 LASSO	67.03	59.74	74.32	.72	56.32	45.07	67.57	.57
REF-SCN-M 100 LASSO	60.73	62.99	58.47	.61	52.09	36.62	67.57	.47
REF-SCN-M 200 LASSO	60.14	48.70	71.58	.64	55.89	23.94	87.84	.59
REF-SCN stacker LASSO	68.11	62.99	73.22	.73	50.60	29.58	71.62	.57
REF-SCN-E 100 PCA	53.69	50.00	57.38	.57	47.84	26.76	68.92	.47
REF-SCN-E 200 PCA	64.74	53.25	76.23	.69	57.38	30.99	83.78	.61
REF-SCN-M 100 PCA	51.93	62.34	41.53	.53	44.07	40.85	47.30	.41
REF-SCN-M 200 PCA	63.17	50.65	75.68	.67	61.55	36.62	86.49	.60
REF-SCN stacker PCA	63.78	53.25	74.32	.68	56.74	32.39	81.08	.63
KLS-SCN-E 100 LASSO	56.88	35.06	78.69	.61	52.87	8.45	97.30	.50
KLS-SCN-E 200 LASSO	64.34	44.81	83.88	.70	55.66	12.68	98.65	.54
KLS-SCN-M 100 LASSO	56.44	46.75	66.12	.58	49.16	25.35	72.97	.50
KLS-SCN-M 200 LASSO	60.55	53.90	67.21	.62	60.26	39.44	81.08	.58
KLS-SCN stacker LASSO	61.70	59.74	63.66	.67	47.95	32.39	63.51	.54
KLS-SCN-E 100 PCA	57.12	57.14	57.10	.59	64.63	54.93	74.32	.46
KLS-SCN-E 200 PCA	60.01	59.09	60.93	.63	50.89	43.66	58.11	.65
KLS-SCN-M 100 PCA	56.15	55.19	57.10	.58	64.07	60.56	67.57	.49
KLS-SCN-M 200 PCA	54.63	53.25	56.01	.61	63.28	54.93	71.62	.64
KLS-SCN stacker PCA	59.24	59.74	58.74	.61	57.24	23.94	90.54	.64
ROI SCN stacker LASSO	69.97	67.53	72.40	.75	59.30	25.35	93.24	.65
ROI SCN stacker PCA	64.43	59.74	69.13	.71	67.10	43.66	90.54	.71

BAC, balanced accuracy; AUC, area under the curve.

### 3.2 Results: classification models

Of all unimodal models trained on the MUC dataset, the LASSO-regularized classifier trained on REF-SCN-E 200 achieved the highest diagnostic performance, with a *BAC* of 67.03% (sensitivity=59.74%, specificity=74.32%, *P*<.001; **Table 2**). However, its external validity in the COBRE sample was limited, showing a *BAC* of 56.32% (*sensitivity*=45.07%, *specificity*=67.56%, *P*=.035; **Table 2**). This model significantly outperformed the other unimodal models, except for the ROI-GMV models, as determined by the Quade’s test (*W* (27, 2673)=29.49, *P*<.001) and *post-hoc* pairwise comparisons. All ROI-GMV based models demonstrated similar training performance without statistically significant differences and maintained their performance on the external validation set (see **Table 2**). When comparing the training performance of unimodal models and multimodal stacked models, the second-level multimodal classifier based on the LASSO-regularized models, performed significantly better than any other models (*BAC*=69.96%, *sensitivity*=67.53%, *specificity*=72.40%, *P*<.001). However, this classifier exhibited no significantly better-than-change generalizability when validated on the COBRE sample (*BAC*=59.30%, *sensitivity*=25.35%, *specificity*=93.24%, *P*=.283). The corresponding ROC curves for the unimodal 200-parcellation models are shown in **Supplementary Figure S4**.

**Figure 1:**
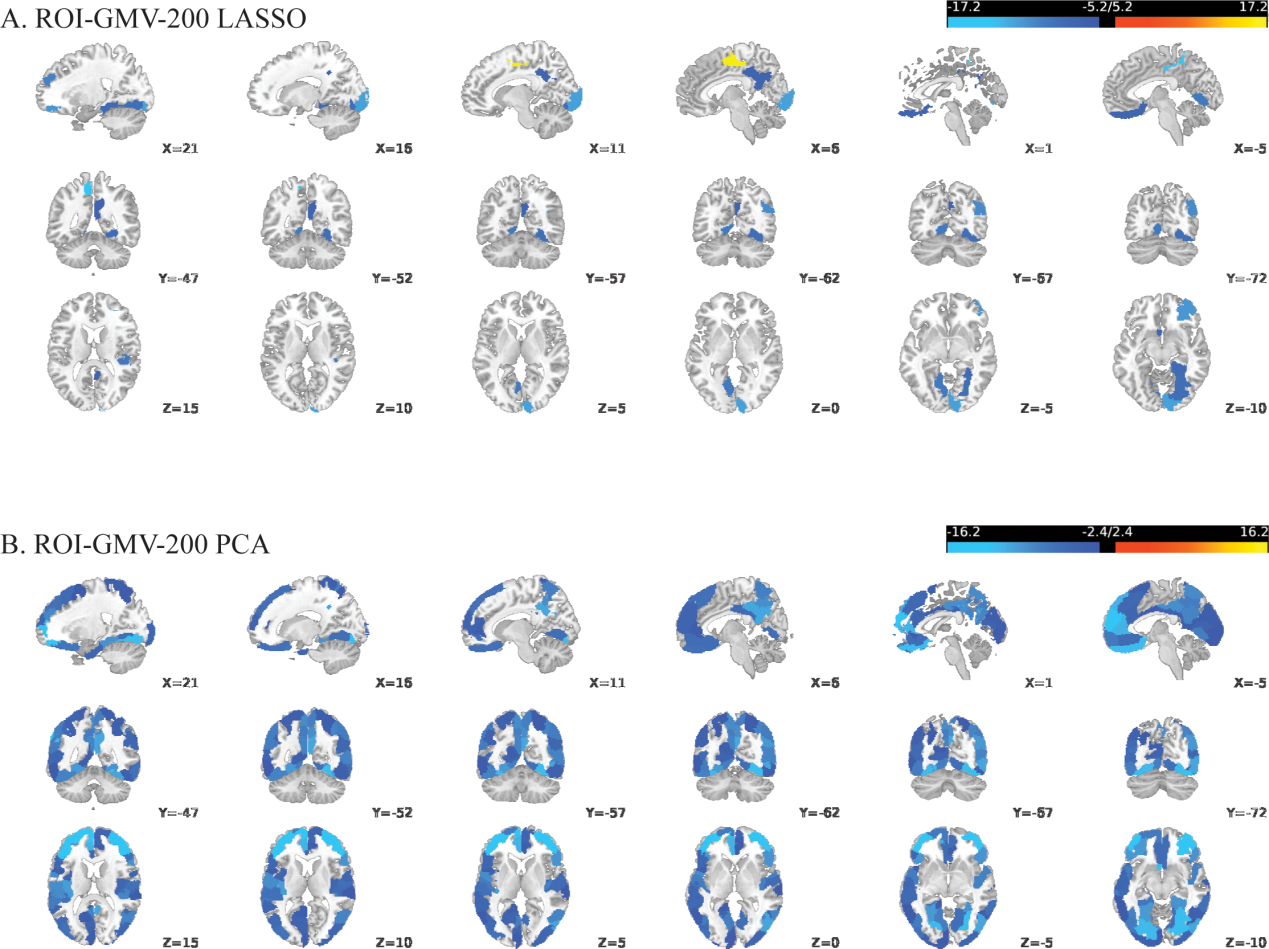
Reliable brain regions for classifying schizophrenia versus controls using two multivariate approaches applied to 200-parcel gray matter volume (GMV) features: **(A)** LASSO-regularized logistic regression with embedded feature selection; **(B)** Principal Component Analysis (PCA). Significant regions are defined using sign-based consistency mapping with FDR-corrected p-values (< 0.05). Colors indicate the median feature weight across cross-validation folds, with blue representing negative weights and yellow representing positive weights.

**Figure 2:**
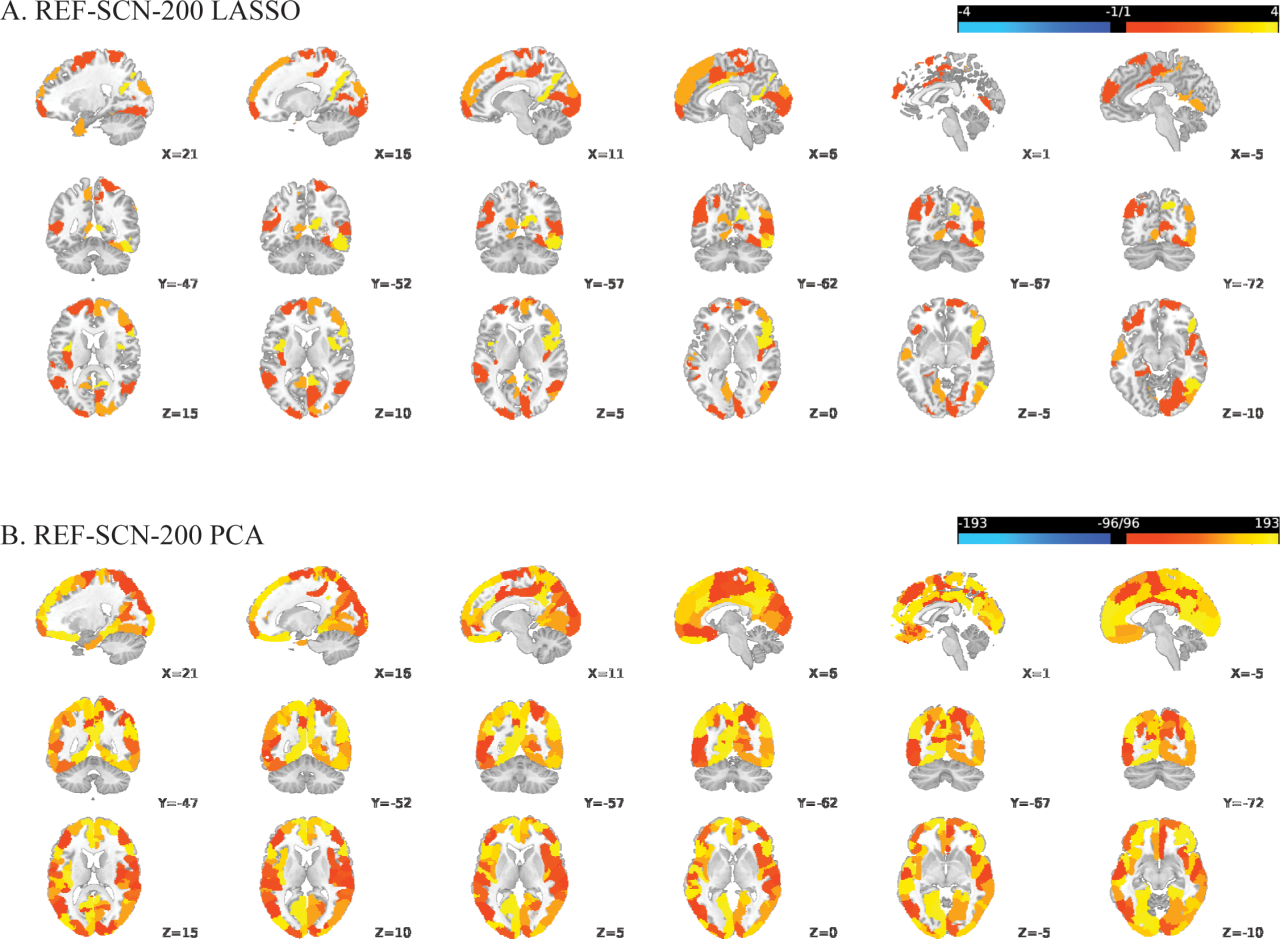
Regional involvement in reliable structural covariance connections predictive of schizophrenia versus controls, based on REF-SCN edge weights derived from a 200-parcel atlas: **(A)** LASSO-regularized feature selection; **(B)** Principal Component Analysis (PCA). Significant connections were identified using sign-based consistency mapping with FDR-corrected p-values (< 0.05). Regional involvement is defined as the absolute number of significant connections per region (nodal degree) and reflected by the color gradient, with brighter shades indicating higher connectivity participation.

For the ROI-GMV-200-LASSO model, sign-based consistency mapping (thresholded at -log10-p>1.3) showed that the GMV of 13 out of the 200 regions in the somatomotor network contributed significantly to the model’s decision function (**Figure 1**). In contrast, 137 out of the 200 regions contributed to the ROI-GMV-200-PCA model’s decision function, mostly including regions in the default mode, frontoparietal, visual, limbic and somatomotor networks (**Figure 1**). *Post-hoc* pairwise comparisons revealed that patients with schizophrenia showed reduced GMV in all these regions compared to HC. For the REF-SCN-E-200-LASSO model and respective PCA-based model, 0.56% and 80.17% of edge weights contributed significantly to the model’s decision function (as determined by sign-based consistency mapping, -log10-p>1.3). In the LASSO model, regions most often connected by edge weight features mapped to the retrosplenial, occipital, and temporal cortices (**Figure 2**). The corresponding PCA model showed lateral and ventrolateral PFC regions to be most important for prediction, as well as some areas of the occipital lobe (**Figure 2**). In the KLS-SCN-E-200-LASSO model and respective PCA-based model, 3.29% and 70.54% of edge weights contributed to models’ predictions. The involved regions were in the PFC, precuneus and the occipital and temporal lobe.

Significant Pearson correlations (FDR-corrected) were observed among the mean decision scores of the unimodal 200-parcellation-based models, as detailed in **Supplementary Table S3**. Notably, looking at the relationship between ROI and SCN models respectively, there was a strong correlation observed between the decision scores of the KLS-SCN-E-200-LASSO and ROI-GMV-200-PCA models (*r* (518)=.94, P<.0001). This high association was driven by high associations in the feature sets before model training; specifically, the first PC of the age and sex-corrected, standardized ROI-GMV-200-PCA features exhibited strong correlations with the respective edges which served as input features in the KLS-SCN-E-200 model (mean *r*[range]=.69 [.31-.90]) but not those of the REF-SCN-E-200 model (mean *r*[range]=.04 [-.15-.21]).

Decision scores of the MUC sample were significantly (FDR-corrected *P<.05*) higher than those in the COBRE sample for 22 of the 28 models (see **Supplementary Table S4**). The cohort effect on decision scores as tested in simple linear regression models whilst controlling for sex and age was significant for 14 of the 28 models (FDR-corrected, see **Supplementary Table S5**).

### 3.3 Results: clinical prediction models

Linear SVR models trained on clinical and sociodemographic data showed the ability to predict the decision scores of the KLS-SCN-E-200 LASSO-regularized model and the ROI-GMV-200 PCA model, but not any of the REF-SCN-based models. The best model performance in terms of R^2^ was observed for the model predicting the decision scores of the KLS-SCN-E 200 LASSO-regularized model (*R^2^ =* 11.01, *MSE*=0.10), and those of the ROI-GMV-200 PCA model (*R^2^ =* 9.01, *MSE*=0.10). For the KLS-SCN-E LASSO decision score model, 54 of the clinical features contributed significantly to the prediction (sign-based consistency mapping), with BMI, and affective negative symptoms assessed with the SANS (precisely, “unchanging facial expression”, “inappropriate affect”, “paucity of expressive gestures”, as well as the summary scores for Affective Flattening or Blunting and Alogia) and the PANSS (precisely, “excitement”, “tension”), and first-generation antipsychotic medication (yes/no, and dosage) contributing most strongly (as defined by cross-validation ratio based on the median). The prediction of the ROI-GMV PCA decision score model was significantly driven by 46 out of the 87 clinical features. The features with the highest cross-validation ratios (median) were the same as those of the KLS-SCN-E-200 LASSO decision score model. The Pearson correlation of the cross-validation ratio (median) feature scores of the two models was *r*=.99 (*P*<.001).

## 4 Discussion

In this study, we assessed the shared and complementary predictive value of cortical structural covariance for the diagnostic classification of patients with schizophrenia. The prediction model trained on the edges of the individual SCNs defined in terms of the contribution to a healthy reference group, achieved the highest classification performance during training and external validation. This model outperformed those trained on individual SCN defined in terms of KL divergence between regional voxel probability density functions. However, it did not show significant improvements over models trained on ROI-GMV. Although a multimodal stacked model performed better during training, it did not generalize to the external validation set.

In line with our expectations, ROI-GMV was informative in diagnosing patients with schizophrenia with a discovery-sample performance of BAC=62.33 – 65.54%. This was consistent across different brain parcellations and dimensionality reduction techniques. The finding that schizophrenia can be detected at the level of the individual from regional morphological information has frequently been reported with models reaching performance accuracies between 60 and 80% (87, 88). Likewise, models trained on individual SCNs were able to classify patients with schizophrenia from HC individuals, consistently across cortical parcellations and dimensionality reduction technique with BAC=60.14 – 68.11% for REF-SCNs and BAC=56.16 – 64.34% for KLS-SCNs. The performance of our KLS-SCN-E models were comparable to those reported by (89) who classified patients with schizophrenia and HC individuals employing edge weights derived from KLS-SCNs in several samples, amongst others, the COBRE sample used as the independent validation set here. They observed highly varying accuracies ranging from 56% to 81.50% (89). One possible explanation for our lower training performance of both ROI-GMV and SCN models might be our focus on cortical regions only. Subcortical regions and their connectivity have previously been shown to be of great importance in the pathophysiology of schizophrenia with studies reporting especially the thalamus and caudate, but also the hippocampus as well as various subcortical areas to have significantly altered connectivity properties (2, 90–95). Additionally, subcortical areas have been proposed to drive fronto-striato-thalamic dysconnectivity in schizophrenia since first episode psychosis patients displayed no significant differences but with ongoing disease thalamic dysconnectivity may become more pronounced (96). Lei et al. (2020) (89) constructed their SCNs based on the automated anatomical labeling (AAL) parcellation atlas, which spans the whole brain. Computing the pairwise similarity between these regions might capture different information compared to our networks defined solely based on the cortical regions of the Schaefer atlases. Here, we focused solely on cortical regions and cortical SCNs because analyses of scanner and scanning protocol differences between the MUC and COBRE samples revealed pronounced effects predominantly in subcortical regions. However, the inclusion of subcortical brain regions and the use of different parcellation schemes or atlases should be assessed in the future (87, 97). Finally, with respect to the SCN models, the choice of engineered features, i.e., edge weights and network metrics, may have caused loss of diagnostic information. The edge-weight-based models may have missed valuable spatial information regarding network topology. Likewise, the network metric computation step involved the selection of network metrics applicable for the properties of brain networks, thereby introducing the possibility of missing valuable information. Our results show that network metrics produce less reliable results than the SCN modalities and are in line with previous reports on graph metrics being less informative than connectome-wide functional connectivity in such classification tasks (2). In this study, we focused on linear ML models, yet, state-of-the-art graph learning methods include kernel-based methods and graph neural networks (GNNs) (98, 99) and might be more suitable for network structured data. While GNNs are increasingly gaining popularity and are increasingly employed to study functional and anatomical connectivity (100–103), there is still limited consensus on how to best apply them to the unique characteristics of brain networks. Considering that training a neural network requires large samples GNNs might in future be especially interesting for individual SCN analysis since sMRI images are typically more easily and efficiently acquired than fMRI and DTI. Here, we opted for linear models due to our limited sample size as well as for straight-forward model explainability.

Our global model explainability results give insights into what regions drove the decisions in our predictive tasks. The identified regions lie in functional networks (104) which have been previously found altered in schizophrenia. Structural alterations in the somatomotor network, the default mode, frontoparietal control, visual and limbic networks informed the decision of the ROI-GMV models. For SCN-based models, the predictive regions mapped to the ventral attention, default mode, frontoparietal control, visual, and somatomotor networks. For REF-SCN, the dorsal attention network was specifically predictive, while for the KLS-SCN, additionally orbital frontal-temporopolar (“limbic” in YEO’s terminology) networks were identified. These findings are in line with findings of widespread GMV loss (105) and disrupted structural covariance (34, 106, 107) and functional connectivity in schizophrenia (92, 94, 96, 108–111). For example (106), found alterations in cortical SCNs especially in PFC regions, while (34) and (107) found reductions in structural integrity of both the frontoparietal and the salience networks but no differences in the dorsal attention, motor and sensory networks. Studies investigating functional connectivity in schizophrenia reported alterations in overall connectivity (110) with regional disruptions being most prominent in the default mode network (reduced and increased connectivity reported) (92, 108, 111), between the dorsal medial PFC and the medial temporal lobe (111), in the frontoparietal network (108), in the sensorimotor cortex, right lateral prefrontal cortex, left insula, and right lingual gyrus (110). Recently, the visual and sensorimotor network have also been implicated in schizophrenia with findings of connector hubs in these regions, as well as in the insula and calcarine (94). While altered connectivity in the frontoparietal network in schizophrenia is consistent across studies, findings are not as clear for the default mode and other networks. Generally, the use of PCA as a dimensionality reduction method resulted in models that were less spatially specific compared to LASSO-regularized models. Additionally, the models based on the same modality (i.e., ROI-GMV, REF-SCN, and KLS-SCN) showed more similarities in significant regions compared to models based on the other modalities.

Our finding that the multimodal stacked generalization model incorporating information from ROI-GMV, REF-SCN, and KLS-SCN outperformed the models trained on one of the modalities alone in terms of discovery performance highlights their complementary diagnostic value. Especially the REF-SCNs seem to capture differential aspects compared to KLS-SCN (E-LASSO) and ROI-GMV (PCA) which resulted in highly associated risk estimates. Further, our findings revealed stronger associations between clinical variables and the derived risk scores of KLS-SCN-E-LASSO and ROI-GMV compared to REF-SCN based models. Taken together, this suggests that KLS-SCN and ROI-GMV features may capture similar disease related information, while deriving REF-SCNs ‘solely’ quantifies whether an individual is similar to the reference group (i.e., has a low deviation) or not (i.e., deviates strongly) whilst discounting information about differential clinical aspects across patients. The role of the reference group’s composition and characteristics should be investigated in the future; capturing the deviation of structural covariance of individuals from reference groups consisting of individuals with different clinical or demographic profiles (instead of healthy individuals) might prove more useful, e.g., in terms of deriving ‘closeness’ scores to different diagnostic brain states (or ‘closeness to younger/older brains’).

Our clinical prediction models revealed that BMI was the strongest predictor of KLS-SCN and ROI-GMV based risk scores for schizophrenia. BMI, an indicator of body fat, has frequently been linked to schizophrenia and both conditions are associated with brain structural changes in many of the same regions. The effects of obesity and schizophrenia appear to be additive, with patients with schizophrenia who have a higher BMI showing more pronounced alterations in brain structure (50). Further, a recent study found disturbed structural covariance between regions in reward and control networks associated with obesity status (112). In general, the ability of SCNs to map individual differences, besides a schizophrenia diagnosis, was investigated by a few studies in various individual prediction tasks. The results mainly suggest limited gained value, e.g., when predicting brain age in adolescents with ADHD and HC individuals (113), or cognitive functioning in patients with Alzheimer’s (114). Increasing efforts are being made with regard to multi-view SCNs combining different morphological modalities when computing the individual SCNs (19, 63) producing promising results, e.g., establishing a link between schizophrenia-related genes and abnormal structural covariance, for brain age prediction (115), and, recently, showing disturbed structural covariance among obese people (112).

We must note a few limitations of our study. First, the patients in the training and validation data exhibited different clinical profiles. The MUC sample included not only patients with chronic schizophrenia but also those at earlier stages of the disorder. This variability could have confounded the algorithm’s decisions, as alterations in brain structure might be less pronounced in patients experiencing a first episode of psychosis. Second, as previously mentioned, our choice of cortical brain parcellations could have led to discarding valuable information. Repeating the analysis with different brain parcellations would further enhance robustness of the findings. Third, we lacked detailed information regarding medication intake beyond whether antipsychotics or antidepressants were used. Including specific substances, duration, and cumulative dosage would have provided more nuanced insights into their (differential) impact on regional brain structure and structural covariance. Additionally, information on patients’ drug consumption was limited to whether they smoked cigarettes and drank alcohol and took any other drugs (yes/no). Including data on which specific other drugs, e.g., cannabis, they used would have been valuable.

In conclusion, despite some open questions and the limitations mentioned above, our study demonstrates that individual SCNs estimated by means of deviation from a healthy reference sample or KL-divergence contain information for the classification of schizophrenia, beyond information contained in ROI-GMV. Whilst only performing modest in terms of classification accuracy, KLS-SCNs effectively captured clinical differences in patients with schizophrenia, primarily driven by BMI and negative symptoms, similar to information extracted by PCA on ROI-GMV features. Therefore, individual SCNs, as a proxy for brain dysconnectivity, may advance the search for biomarkers of schizophrenia.

## Data Availability

The original contributions presented in the study are included in the article/**Supplementary Material**. Further inquiries can be directed to the corresponding author.

## The PRONIA consortium

The following authors participated in the screening, recruitment, rating, examination, and follow-up of the study participants used in the current analyses. The authors are listed in alphabetical order according to the institution of affiliation.

Department of Psychiatry and Psychotherapy, Ludwig-Maximilian-University, Munich, Germany: Shalaila Haas, Alkomiet Hasan, Claudius Hoff, Ifrah Khanyaree, Camilla Krämer, Aylin Melo, Susanna Muckenhuber-Sternbauer, Yanis Köhler, Oemer Faruk Oeztuerk, Nora Penzel, David Popovic, Adrian Rangnick, Sebastian von Saldern, Rachele Sanfelici, Moritz Spangemacher, Ana Tupac, Maria Fernanda Urquijo, Johanna Weiske, Antonia Wosgien.

Department of Psychiatry and Psychotherapy, University of Cologne, Cologne, Germany: Karsten Blume, Dennis Hedderich, Dominika Julkowski, Nathalie Kaiser, Thorsten Lichtenstein, Ruth Milz, Alexandra Nikolaides, Tanja Pilgram, Mauro Seves, Martina Wassen.

Department of Psychiatry (Psychiatric University Hospital, UPK), University of Basel, Switzerland: Christina Andreou, Laura Egloff, Fabienne Harrisberger, Ulrike Heitz, Claudia Lenz, Letizia Leanza, Amatya Mackintosh, Renata Smieskova, Erich Studerus, Anna Walter, Sonja Widmayer.

Institute of Mental Health & School of Psychology, University of Birmingham, United Kingdom: Chris Day, Sian Lowri Griffiths, Mariam Iqbal, Mirabel Pelton, Pavan Mallikarjun, Alexandra Stainton, Ashleigh Lin.

Department of Psychiatry, University of Turku, Finland: Alexander Denissoff, Anu Ellilä, Tiina From, Markus Heinimaa, Tuula Ilonen, Päivi Jalo, Heikki Laurikainen, Antti Luutonen, Akseli Mäkela, Janina Paju, Henri Pesonen, Reetta-Liina Säilä, Anna Toivonen, Otto Turtonen.

Department of Psychiatry (Psychiatric University Hospital LVR/HHU Düsseldorf), University of Düsseldorf, Germany: Sonja Botterweck, Norman Kluthausen, Gerald Antoch, Julian Caspers, Hans-Jörg Wittsack.

Department of Basic Medical Science, Neuroscience and Sense Organs - University of Bari Aldo Moro: Giuseppe Blasi, Giulio Pergola, Grazia Caforio, Leonardo Fazio, Tiziana Quarto, Barbara Gelao, Raffaella Romano, Ileana Andriola, Andrea Falsetti, Marina Barone, Roberta Passiatore, Marina Sangiuliano.

Department of Psychiatry and Psychotherapy of the University of Münster, Germany: Marian Surmann, Olga Bienek, Udo Dannlowski.

General Electric Global Research Inc., USA.

Ana Beatriz Solana, Manuela Abraham, Timo Schirmer.

Workgroup of Paolo Brambilla, University of Milan, Italy:

Department of Neuroscience and Mental Health, Fondazione IRCCS Ca’ Granda Ospedale Maggiore Policlinico, University of Milan, Milan, Italy: Carlo Altamura, Marika Belleri, Francesca Bottinelli, Adele Ferro, Marta Re

Programma2000, Niguarda Hospital, Milan: Emiliano Monzani, Maurizio Sberna

San Paolo Hospital, Milan: Armando D’Agostino, Lorenzo Del Fabro

Villa San Benedetto Menni, Albese con Cassano (CO): Giampaolo Perna, Maria Nobile, Alessandra Alciati

Workgroup of Paolo Brambilla at the University of Udine, Italy.

Department of Medical Area, University of Udine, Udine, Italy: Matteo Balestrieri, Carolina Bonivento, Giuseppe Cabras, Franco Fabbro.

IRCCS Scientific Institute “E. Medea”, Polo FVG, Udine: Marco Garzitto, Sara Piccin.
