# Supplementary material for: Exploring the predictive value of structural covariance networks for the diagnosis of schizophrenia
Source: Front Psychiatry. 2025 Jun 9;16:1570797. doi: 10.3389/fpsyt.2025.1570797 (PMC12183260; doi:10.3389/fpsyt.2025.1570797)
Supplement: Supplementary file 1 [file DataSheet1.pdf]

Supplementary Material

1 Supplementary Methods

1.1 Neuroimaging acquisition parameters

Table S1. Neuroimaging acquisition parameters for the structural MRI data across MUC, COBRE, the different PRONIA sites, and OASIS-3.

Site	Scanner Model	Field Strength	Flip Angle	Coil Channels	Voxel Size (mm)	TR (ms)	TE (ms)	FOV (mm)	Number of slices
MUC	SIEMENS MAGNETOM VISION	1.5T	12	8	0.45 × 0.45 × 1.5	11.6	4.9	230x 230	126
COBRE	SIEMENS TIM	3T	7	12	1x1x1	2530	1.64, 3.5, 5.36, 7.22, 9.08	256x 256	?
PRONIA									
Munich	Philips Ingenia	3T	8	32	0.97 x 0.97 x 1	9.5	5.5	250 x 250	190
Milan	Philips Achieva Intera	1.5T	12	8	0.93 x 0.93 x 1	Short - est (8.1)	Short - est (3.7)	240 x 240	170
Cologne	Philips Achieva	3T	8	8	0.97 x 0.97 x 1	9.5	5.5	250 x 250	190
Basel	SIEMENS Verio	3T	8	12	1 x 1 x 1	2000	3.4	256 x 256	176

Birmingham	Philips Achieva	3T	8	32	1 x 1 x 1	8.4	3.8	288 x 287	175
Turku	Philips Ingenuity	3T	7	32	1 x 1 x 1	8.1	3.7	256 x 256	176
Udine	Philips Achieva	3T	12	8	0.93 x 0.93 x 1	Short - est (8.1)	Short - est (3.7)	240 x 240	170
Münster	SIEMENS Prisma fit	3T	8	12	1 x 1 x 1	2130	2.3	256 x 256	192
OASIS-3									
	SIEMENS TrioTim	3T	8	20	1 x 1 x 1	2.4	3.2	256* 256	
	BioGraph PET-MR	3T	9	20	1 x 1 x 1	2.3	2.9	256* 256	

1.2 Preprocessing of structural MRI data

Initially, a denoising phase was conducted utilizing Spatially Adaptive Non-Local Means (SANLM) filtering. This was followed by intra-subject bias field correction to account for low-frequency intensity inhomogeneities. Subsequently, segmentation was performed employing an Adaptive Maximum A Posteriori (AMAP) technique [61], which includes global intensity normalization and ensures uniform segmentation across cortical and subcortical structures. This was followed by a second denoising step employing a Markov Random Field approach, integrating spatial prior information into the AMAP-generated segmentation. Next, a Local Adaptive Segmentation (LAS) step was employed to mitigate WM inhomogeneities and varying GM intensities. This step further refined intensity normalization by correcting local intensity inhomogeneities, particularly in WM and GM. Further, a Partial Volume Segmentation algorithm was implemented to model tissues between GM and WM, and between GM and cerebrospinal fluid (CSF), applied to AMAP-generated tissue segments. Subsequently, a high-dimensional DARTEL registration [62] was executed, aligning the image with an MNI-template derived from MRI data of 555 healthy controls in the IXI database (http://www.braindevelopment.org). Following registration, GM maps were multiplied with Jacobian

determinants obtained during registration to yield GM volume maps. Finally, the CAT12 Quality Assurance framework was utilized to assess the quality of GMV maps.

1.3 Structural similarity networks: Reference group approach (REF-SCN)

Before deriving the reference group -based SCNs (REF-SCNs), we accounted for systematic differences between the images across the different sites of the PRONIA sample and the OASIS-3 cohort. For each ROI, we subtracted the difference of a site's mean GMV and the overall mean GMV. To additionally account for non-linear effects of site, we performed independent component analysis using the fastICA algorithm [78], and subsequently only projected the independent components not correlated with site back to the original feature space. For the computation of the REF-SCN networks, we further calibrated the training and validation dataset to the reference sample by subtracting the difference of a cohort mean GMV and the mean GMV of the reference sample for each ROI.

From the harmonized data, we followed a two-step procedure to obtain an individual REF-SCN (see Figure S1):

First, a group connectivity matrix was computed by calculating the Pearson's correlation coefficient between the mean GMV of ROI pairs of a parcellation across healthy controls in the reference sample. The resulting correlation matrix represents the P x P weighted connectivity matrix of the reference SCN (P = number of parcels).

Second, following a method first introduced by [2], the individual contribution of subject i was quantified as the change in the group structural covariance network when subject i was added to the reference group prior to computing the group connectivity matrix. Thus, the connectivity matrix C_i of a subject i's SCN is defined as

$$C_i = \mathrm{cor}(C_{ref+i}) - \mathrm{cor}(C_{ref}),$$

(1)

where C_{ref+i} is the group Pearson correlation matrix of the reference group including the subject i's regional GMV data and C_{ref} the group Pearson correlation matrix of only the reference group.

C_i represents the connectivity matrix of the individual SCN of subject i. Each ROI of the respective parcellation corresponds to a node in the SCN, which each are connected by edges. Edge weights represent the change subject i imposes on the connectivity of the reference network of reference HCs. SCNs were computed for two parcellation atlases comprised of 100 and 200 parcels respectively [3].

The resulting individual REF-SCNs represent the individual contribution of a subject to the group structural covariance network of the healthy reference group.

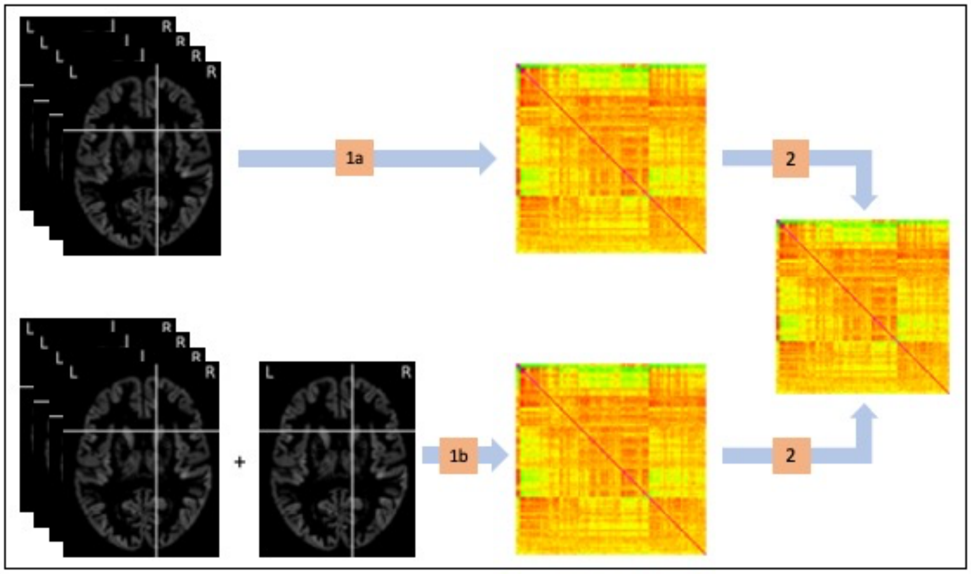

Figure S1. General schema of network construction. (1a) Reference group network: Atlas-based parcellation of GMV maps & Pearson correlation between regional mean GMV across individuals. (1b) Reference group + individual network: Atlas-based parcellation of GMV maps & Pearson correlation between regional mean GMV across individuals. (2) Computation of differences between (1a) and (1b) & thresholding resulting in individual‘s connectivity matrix.

1.4 Structural covariance networks: KL-divergence approach (KLS-SCN)

The general network construction workflow is visualized in Fig. S2 (adapted from [4]). To define the nodes of the networks, we parcellated each processed T1-weighted image into 100 and 200 ROIs respectively using the same atlases as for the ROI GMV and REF-SCN features. In the KL-divergence based SCNs (KLS-SCN), edges represented the structural similarity between the pair of connected regions. We quantified this similarity employing a symmetric form of the Kulback-Leibler divergence (KLS) between their respective probability density functions (PDF) of voxel-wise GMV. A PDF describes all possible values and likelihoods a variable can take within a specified range. It is semi-positively defined, and the area under the PDF's graph sums to 1. The KL divergence D_{KL} is a concept from probability and information theory that measures how a probability distribution p(x) differs from another probability distribution q(x). It measures the information lost when p(x) is approximated using q(x) and is formally defined as

Where p(x) and q(x) are two PDFs of a discrete random variable x. Since the original form of the KL divergence is not symmetrical, and

$$\mathrm{KLS}(p(x), q(x)) \ = \ e^{-D_{KL}(p(x), q(x))},$$

(2)

Here, we used a variation of the KL divergence

$$D_{KL}(p(x), q(x)) = \sum_{x \in X} (p(x) \ln \frac{p(x)}{q(x)} + q(x) \ln e^{-D_{KL}(p(x),q(x))},$$

(3)

Then, the KLS was computed as

$$KLS(p(x), q(x)) = e^{-D_{KL}(p(x),q(x))},$$

(4)

Ranging from 0 to 1 where a value of 1 indicated identical distributions. These computations resulted in a morphological connectivity matrix (100x100 or 200x200) per subject. To estimate the regional PDFs of the voxel-wise GMVs, we used kernel density estimations (KDE) with automatically chosen bandwidth using the density function in R (version 4.2.2) [5].

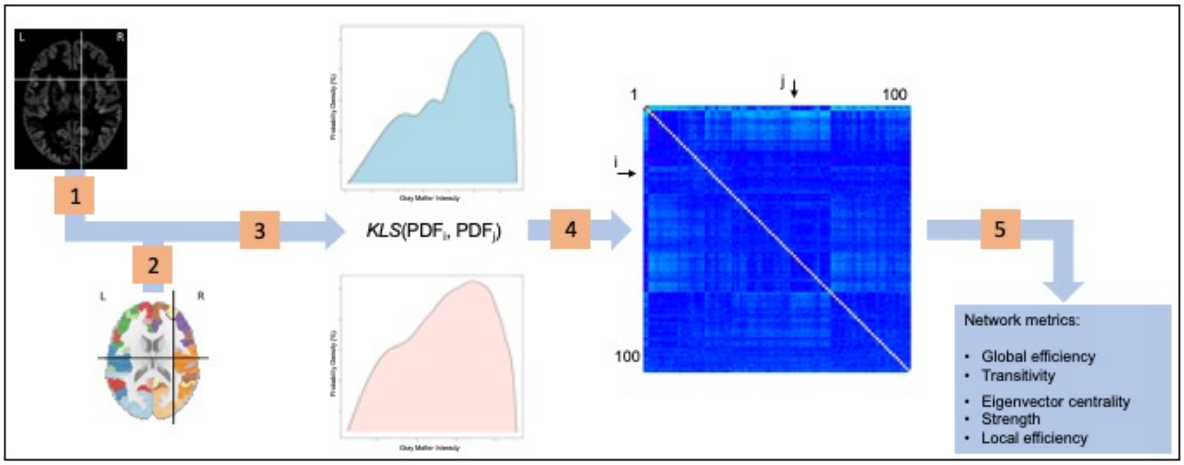

Figure S2. General schema of network construction (adapted from [4]). (1) Preprocessing of obtained MRI images and GMV estimation. (2) Brain parcellation using a brain atlas (depicted: Schaefer atlas with 100 parcels). (3) Computation of the similarity matrix by calculating the similarity between distributions of the respective parcels. (4) Resulting similarity matrix. (5) Calculation of various network metrics.

1.5 Networks metrics

While both approaches have been proven to perform well for brain network analysis [6], [7], they both reduce the information available in networks. Network metrics capture the network's topology at the global and node level, whereas vectorized connectivity matrices preserve the information contained in edge weights. To gain insights into various aspects of the network properties, we chose the following common metrics appropriate for our weighted, undirected, and fully connected SCNs: Global efficiency, transitivity, eigenvector centrality, strength, and local efficiency. *Global Efficiency* is defined as the inverse average shortest path length, is thus related to the characteristic path length, and reflects the capacity for network-wide communication. It is therefore a measure of functional

integration [8], [9]. *Transitivity* (global metric) is based on the number of triangles of nodes in a network. One may consider it a variant of the clustering coefficient, reflecting the formation of clusters of brain regions. Transitivity thus describes a network's functional segregation, i.e., specialized neural processing in certain regions [9]–[11]. *Eigenvector centrality* is a local metric measuring a node's centrality in the sense that both the number and quality of connections to immediately neighbouring nodes is counted. Well-connected nodes thus achieve higher values [10], [12]. *Strength* (local metric) is the weighted variant of the metric degree and has been defined by a node's sum of all neighbouring link weights [11]. *Local efficiency* is a metric which represents functional segregation, but also "fault tolerance" on the local level [9], [13], [14].

1.6 Classification models

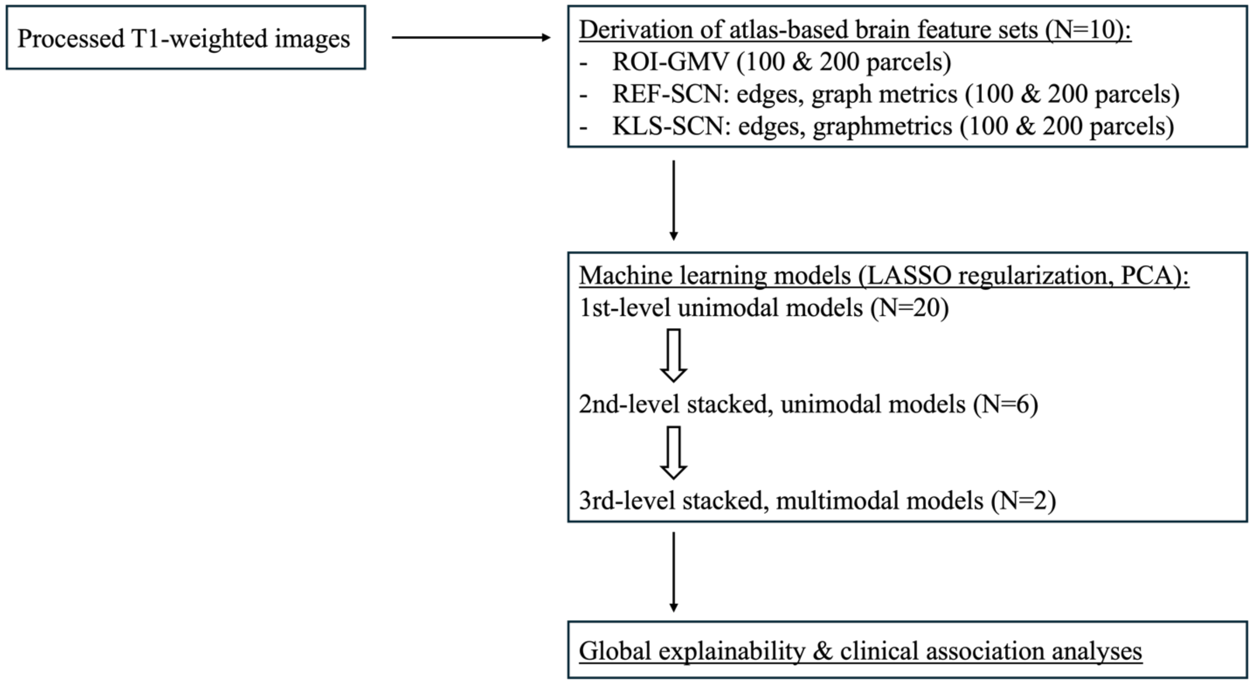

Figure S3. Flowchart of analysis pipeline. Processed T1-weighted images were used to derive ROI-GMV and SCN feature sets based on the Schaefer atlases with 100 and 200 parcels. These served as input for the machine learning models. Finally, explainability and posthoc association analyses were performed.

1.7 Clinical prediction models

1.7.1 Model pipelines:

The clinical prediction models were built and trained within NeuroMiner using a 10-by-10 nested cross-validation structure with 10 outer permutations. Preprocessing of the features included scaling (from 0-1), imputation of missing values with the median of the 5 nearest neighbors using a sequential k-nearest neighbor (SeqkNN) algorithm (https://www.mathworks.com/matlabcentral/fileexchange/60128-sequential-knn-imputation-method), soft feature selection by upweighting those features ranked in terms of Pearson correlation coefficient

with the target label (in descending order) with an exponential multiplier optimized in the range of 0-3, dimensionality reduction by performing principal component analysis (PCA) and extracting between 3 and 21 (optimized) of the principal components (PC), and standardization using the median. For learning, we used an L2-regularized L2-loss support vector regressor (SVR) model from the LIBLINEAR implementation, with tolerance = 0.01, and c was optimized between 0.05 and 0.5. All preprocessing steps, as well as hyperparameter optimization were performed within the cross-validation loops. To assess feature importance, the same procedure as for the binary classification models was employed (see main manuscript).

1.7.2 Input features:

- Stage(?)
- Age
- Sex
- Handedness
- BMI
- Education [years]
- Illness duration [years]
- Age of onset [COMP??]
- Number of hospitalizations
- Antipsychotic medication [yes/ no]
- Antidepressant medication [yes/ no]
- TOTAL_AD
- TYPICAL_ATYPICAL_YESNO
- TYPICAL_AP
- ATYPICAL_AP
- TOTAL_AP
- CPZ_EQ
- PANSS items: P1, P2, P3, P4, P5, P6, P7, N1, N2, N3, N4, N5, N6, N7, G1, G2, G3, G4, G5, G6, G7, G8, G9, G10, G11, G12, G13, G14, G15, G16
- PANSS total score
- PANSS Positive score
- PANSS Negative score
- PANSS_General score

- SANS items: S1, S2, S3, S4, S5, S6, S7, S8, S9, S10, S11, S12, S13, S14, S15, S16, S17, S18, S19, S20, S21, S22, S23, S24
- SANS_Affective Flattening
- SANS_Alogia
- SANS_Avolition
- SANS_Anhedonia
- SANS_Attention
- SANS total score
- Nicotine
- Alcohol group
- Drugs
- Duration of untreated psychosis

2 Results

2.1 Sociodemographic characteristics of the reference sample

Table S2.

Sociodemographic characteristics of the reference sample.

Variable	Reference sample
Mean age in years (SD)	35.6 (20.1)
N sex female (%)	341 (54.4)
Mean years in education (SD)	15.8 (3.2)
Mean schizophrenia score (SD)	-0.4 (0.8)
N Cohorts (%)	
OASIS-3	138 (22.0)
PRONIA	489 (78.0)
N PRONIA sites (%)	
University of Munich	117 (18.7)
University of Milan	36 (5.7)
University of Basel	73 (11.6)
University of Cologne	76 (12.1)
University of Birmingham	56 (8.9)
University of Turku	56 (8.9)
University of Udine	75 (12.0)

Note. SD = standard deviation.

2.2 Classification model results

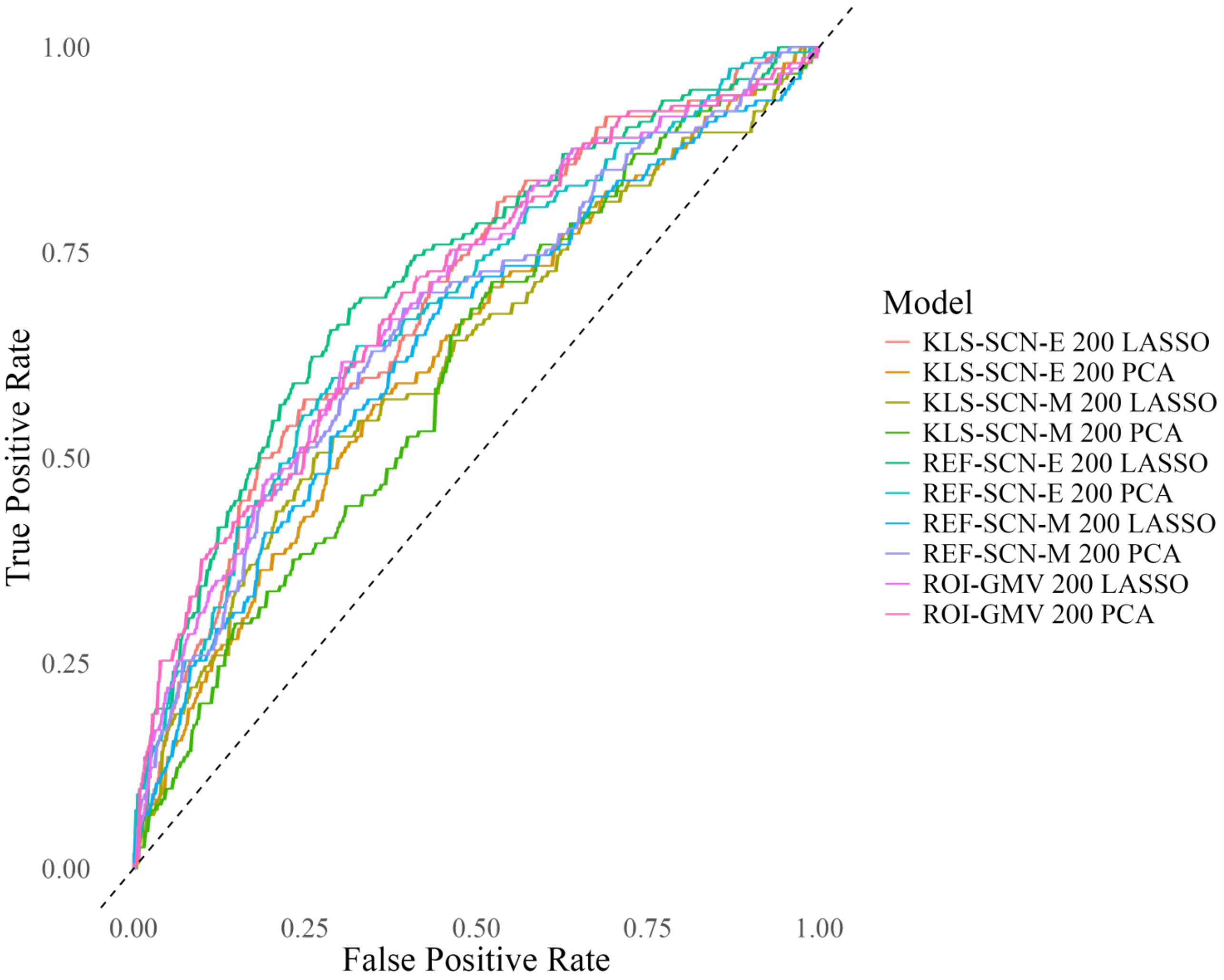

Figure S4. Receiver Operating Characteristic (ROC) Curves for Unimodal 200-Parcel Models. The ROC curves illustrate the performance of each model in distinguishing between [state the classes, e.g., patients vs. controls], with key metrics such as AUC provided in the inset.

Table S3.

Significant Pearson correlations between the mean decision scores of the unimodal, 200-parcel models.

	ROI L.	ROI P.	REF E L.	REF E P.	KLS E L.	KLS E P.	REF M L.	REF M P.	KLS M L.	KLS M P.
ROI LASSO										
ROI PCA	.81									
REFE LASSO	.18	.21								
REFE PCA	.23	.29								

KLSE LASSO	.73	.94	.19	.29					
KLSE PCA	.17	.19	.99		.18				
REFM LASSO	.22	.25			.25				
REFM PCA	.94	.88	.20	.25	.82	.18	.25		
KLSM LASSO	.15	.15	.33		.15	.35		.15	
KLSM PCA	.59	.60	.17	.40	.56	.17	.38	.62	.22

Note. REFE = REFSCN edges; REFM = REFSCN metrics; KLSE = KLSSCN edges; KLSM = KLSSCN metrics; L = LASSO; P = PCA.

Table S4.

Cohort group comparisons decision scores

ID	t	df	p	mean_muc	mean_cobre	difference	p_fdr
ROI-GMV-100-LASSO-SVC2	2.75	245.19	0.006	-0.08	-0.17	0.1	0.01
ROI-GMV-100-L2SVC	1.83	241.09	0.069	-0.05	-0.1	0.05	0.081
ROI-GMV-200-LASSO-SVC2	3.4	228.59	0.001	-0.09	-0.23	0.14	0.002
ROI-GMV-200-L2SVC	2.51	230.45	0.013	-0.07	-0.15	0.08	0.018
ROI-GMV stacker	3.22	234.52	0.001	-0.04	-0.13	0.09	0.003
ROI-GMV stacker L2SVC	2.45	233.34	0.015	-0.04	-0.11	0.07	0.02
REF-SCN-E-100-LASSO-SVC2	7.13	292.25	0	-0.04	-0.18	0.14	0
REF-SCN-E-100-L2SVC	3.8	224.2	0	-0.04	-0.11	0.07	0.001
REF-SCN-E-200-LASSO-SVC2	-1.57	267.79	0.116	-0.1	-0.04	-0.06	0.121

REF-SCN-E-200-L2SVC	2.96	236.28	0.003	-0.05	-0.15	0.09	0.006
REF-SCN-M-100-LASSO-SV	4.11	228.05	0	-0.01	-0.06	0.05	0
REF-SCN-M-100-PCA-L2SVC	3.78	218.51	0	0	-0.02	0.02	0.001
REF-SCN-M-200-LASSO-SV	2.34	499.79	0.02	-0.13	-0.26	0.12	0.025
REF-SCN-M-200-PCA-L2SVC	2.77	532.02	0.006	-0.02	-0.14	0.12	0.01
REF-SCN stacker	2.78	246.82	0.006	-0.06	-0.15	0.09	0.01
REF-SCN stacker L2SVC	3.1	241.26	0.002	-0.04	-0.12	0.08	0.005
KLS-SCN-E-100-LASSO-SVC2	8.04	236.08	0	-0.49	-1.03	0.54	0
KLS-SCN-E-100-L2SVC	3.55	239.84	0	-0.02	-0.08	0.06	0.001
KLS-SCN-E-200-LASSO-SVC2	7.91	255.76	0	-0.54	-1.04	0.5	0
KLS-SCN-E-200-L2SVC	1.66	305.7	0.097	-0.03	-0.06	0.03	0.105
KLS-SCN-M-100-LASSO-SV	3.65	206.28	0	-0.13	-0.28	0.15	0.001
KLS-SCN-M-100-PCA-L2SVC	1.96	179.68	0.052	-0.02	-0.07	0.05	0.063
KLS-SCN-M-200-LASSO-SV	2.67	255.93	0.008	-0.14	-0.25	0.11	0.012

KLS-SCN-M-200-PCA-L2SVC	0.68	250.05	0.497	-0.02	-0.04	0.01	0.497
KLS-SCN stacker	8.72	281.54	0	-0.04	-0.24	0.2	0
KLS-SCN stacker L2SVC	1.72	301.31	0.087	-0.02	-0.04	0.03	0.097
Multimodal stacker	7.04	272.36	0	-0.07	-0.29	0.22	0
Multimodal stacker L2SVC	3.05	247.51	0.003	-0.05	-0.13	0.08	0.005

Table S5.

Simple linear regression models to determine cohort effect on decision scores while controlling for age and sex.

ID	r.sqrt	adj.r.sqrt	cohort_estimate	cohort_t	age_estimate	age_t	sex_estimate	sex_t	p_fdr	cohort_p_fdr	age_p_fdr	sex_p_fdr
ROI-GMV-100-LASSO-SVC2	0.02	0.01	-0.10	-2.73	0.00	-1.06	-0.07	-2.08	0.052	0.083	1.000	0.488
ROI-GMV-100-L2SVC	0.01	0.00	-0.05	-1.85	0.00	-0.13	-0.02	-0.95	0.764	0.432	1.000	1.000
ROI-GMV-200-LASSO-SVC2	0.04	0.03	-0.14	-3.52	0.00	-2.07	-0.10	-3.02	0.000	0.009	0.625	0.044
ROI-GMV-200-L2SVC	0.02	0.01	-0.09	-2.59	0.00	-0.93	-0.05	-1.86	0.097	0.097	1.000	0.708

ROI-GMV stacker	0.03	0.02	-0.10	-3.33	0.00	-1.31	-0.06	-2.67	0.003	0.017	1.000	0.116
ROI-GMV stacker L2SVC	0.02	0.01	-0.07	-2.52	0.00	-0.80	-0.04	-1.82	0.127	0.107	1.000	0.708
REF-SCN-E-100-LASSO-SVC2	0.12	0.12	-0.13	-5.90	0.00	-5.75	-0.07	-3.50	0.000	0.000	0.000	0.010
REF-SCN-E-100-L2SVC	0.08	0.07	-0.06	-3.03	0.00	-6.24	-0.01	-0.43	0.000	0.039	0.000	1.000
REF-SCN-E-200-LASSO-SVC2	0.08	0.08	0.04	1.00	0.00	-3.32	-0.22	-6.34	0.000	0.634	0.018	0.000
REF-SCN-E-200-L2SVC	0.03	0.03	-0.12	-3.57	0.00	1.11	-0.10	-3.63	0.001	0.008	1.000	0.006
REF-SCN-M-100-LASSO-SV	0.07	0.07	-0.04	-3.11	0.00	-5.64	0.01	1.23	0.000	0.031	0.000	1.000
REF-SCN-M-100-PCA-L2SVC	0.07	0.07	-0.01	-2.68	0.00	-5.16	0.02	3.34	0.000	0.084	0.000	0.016
REF-SCN-M-200-LASSO-SV	0.02	0.01	-0.12	-1.53	-0.01	-2.16	-0.12	-1.81	0.095	0.525	0.525	0.708
REF-SCN-M-200-PCA-L2SVC	0.01	0.00	-0.13	-1.87	0.00	0.15	-0.01	-0.19	0.764	0.432	1.000	1.000
REF-SCN stacker	0.11	0.10	-0.09	-2.89	-0.01	-5.21	-0.17	-6.30	0.000	0.055	0.000	0.000

REF-SCN stacker L2SVC	0.04	0.03	-0.10	-3.63	0.00	0.66	-0.09	-3.84	0.000	0.007	1.000	0.003
KLS-SCN-E-100-LASSO-SVC2	0.19	0.19	-0.56	-8.45	-0.01	-5.34	-0.39	-7.09	0.000	0.000	0.000	0.000
KLS-SCN-E-100-L2SVC	0.02	0.02	-0.06	-3.36	0.00	-1.47	-0.02	-1.33	0.012	0.016	1.000	1.000
KLS-SCN-E-200-LASSO-SVC2	0.17	0.17	-0.53	-8.03	-0.01	-4.37	-0.40	-7.22	0.000	0.000	0.000	0.000
KLS-SCN-E-200-L2SVC	0.01	0.00	-0.03	-1.54	0.00	-0.71	-0.03	-1.83	0.486	0.525	1.000	0.708
KLS-SCN-M-100-LASSO-SV	0.04	0.04	-0.15	-3.93	0.00	-2.59	-0.08	-2.58	0.000	0.002	0.176	0.140
KLS-SCN-M-100-PCA-L2SVC	0.01	0.01	-0.05	-2.36	0.00	-1.12	-0.01	-0.81	0.263	0.148	1.000	1.000
KLS-SCN-M-200-LASSO-SV	0.03	0.03	-0.12	-2.74	0.00	-1.62	-0.13	-3.48	0.001	0.083	1.000	0.010
KLS-SCN-M-200-PCA-L2SVC	0.00	0.00	-0.02	-0.80	0.00	0.04	-0.02	-1.11	0.764	0.634	1.000	1.000
KLS-SCN stacker	0.19	0.18	-0.21	-8.38	0.00	-4.81	-0.15	-7.48	0.000	0.000	0.000	0.000
KLS-SCN stacker L2SVC	0.01	0.01	-0.03	-1.62	0.00	-0.61	-0.03	-1.89	0.486	0.525	1.000	0.708
Multimodal stacker	0.18	0.17	-0.23	-6.94	-0.01	-5.47	-0.21	-7.70	0.000	0.000	0.000	0.000

Multimodal stacker L2SVC	0.03	0.02	-0.09	-3.21	0.00	-0.50	-0.07	-2.88	0.008	0.024	1.000	0.066
